# Construction and validation of risk prediction models for pulmonary embolism in hospitalized patients based on different machine learning methods

**DOI:** 10.3389/fcvm.2024.1308017

**Published:** 2024-06-25

**Authors:** Tao Huang, Zhihai Huang, Xiaodong Peng, Lingpin Pang, Jie Sun, Jinbo Wu, Jinman He, Kaili Fu, Jun Wu, Xishi Sun

**Affiliations:** ^1^ Emergency Medicine Center, Affiliated Hospital of Guangdong Medical University, Zhanjiang, Guangdong, China; ^2^ Respiratory and Critical Care Medicine, Affiliated Hospital of Guangdong Medical University, Zhanjiang, Guangdong, China

**Keywords:** pulmonary embolism, hospitalized patients, machine learning, prediction models, random forest

## Abstract

**Objective:**

This study aims to apply different machine learning (ML) methods to construct risk prediction models for pulmonary embolism (PE) in hospitalized patients, and to evaluate and compare the predictive efficacy and clinical benefit of each model.

**Methods:**

We conducted a retrospective study involving 332 participants (172 PE positive cases and 160 PE negative cases) recruited from Guangdong Medical University. Participants were randomly divided into a training group (70%) and a validation group (30%). Baseline data were analyzed using univariate analysis, and potential independent risk factors associated with PE were further identified through univariate and multivariate logistic regression analysis. Six ML models, namely Logistic Regression (LR), Decision Tree (DT), Random Forest (RF), Naive Bayes (NB), Support Vector Machine (SVM), and AdaBoost were developed. The predictive efficacy of each model was compared using the receiver operating characteristic (ROC) curve analysis and the area under the curve (AUC). Clinical benefit was assessed using decision curve analysis (DCA).

**Results:**

Logistic regression analysis identified lower extremity deep venous thrombosis, elevated D-dimer, shortened activated partial prothrombin time, and increased red blood cell distribution width as potential independent risk factors for PE. Among the six ML models, the RF model achieved the highest AUC of 0.778. Additionally, DCA consistently indicated that the RF model offered the greatest clinical benefit.

**Conclusion:**

This study developed six ML models, with the RF model exhibiting the highest predictive efficacy and clinical benefit in the identification and prediction of PE occurrence in hospitalized patients.

## Introduction

Venous thromboembolism (VTE), including deep vein thrombosis (DVT) and pulmonary embolism (PE), is a common and life-threatening condition (1). PE is a general term for a group of diseases or clinical symptoms caused by various emboli from different sources and types (thrombus, fat embolus, amniotic fluid embolus, air embolus, etc.) entering the main pulmonary artery and its branches, thus causing pulmonary artery stenosis or even occlusion. PE is categorized into several types based on different sources of emboli, including pulmonary thromboembolism (PTE), fat embolism syndrome, amniotic fluid embolism, air embolism, etc. Among these, PTE is the most frequently observed (2).

PE ranks as the third leading cause of cardiovascular mortality globally, following stroke and myocardial infarction (3). In the United States, approximately 360,000 people are diagnosed with PE each year, and approximately 60,000–100,000 patients die from PE each year (4). However, as a cardiopulmonary vascular disease with high morbidity and mortality, most patients with PE have diverse and atypical clinical symptoms, and some routine examinations lack specificity, resulting in high rates of clinical misdiagnosis and underdiagnosis. Once diagnosed accurately at an early stage and given appropriate treatment, the morbidity and mortality of PE can be effectively reduced. Therefore, early diagnosis of PE and timely assessment of the risk of PE are crucial. Due to the lack of specificity in the clinical symptoms of PE, patients’ lack of attention, the absence of hospital instruments and equipment, and the limited knowledge of medical workers, the screening and diagnosis of PE become challenging.

Although some scoring rules have been developed for assessing the risk of PE, such as the Wells score and the revised Geneva score, which are often used in clinical practice, they are mainly designed for outpatients with suspected PE and are inaccurate for use in hospitalized patients (5, 6). Consequently, there is a need to develop a predictive tool with high efficacy specifically for assessing the risk of PE in hospitalized patients.

In recent years, significant strides in computer performance have led to a notable phenomenon of medical-industrial integration, marked by the gradual introduction of machine learning algorithms into the medical domain. Machine learning (ML) have been extensively employed in the detection and diagnosis of various diseases, including heart disease (7), kidney disease (8), liver disease (9), and diabetes (10). Furthermore, the utilization of ML in PE is prevalent. For instance, Villacorta et al. employed ML to investigate the role of D-dimer in risk stratification of PE (11). Su et al. devised a ML technique to discern the severity of PE using clinical features and hematological indicators (12). Wang et al. utilized ML to forecast the 30-day mortality rate of critically ill PE patients (13). These studies underscore the promising application of ML techniques, developed from high-dimensional medical data, for PE. Notably, there remains a dearth of attention on the development and utilization of machine learning to identify the risk of PE in hospitalized patients. Consequently, the objective of this study was to employ different ML methods to construct risk prediction models for PE in hospitalized patients and to assess and compare the predictive efficacy and clinical benefit of each model, aiming to determine the optimal model.

## Patients and methods

### Study design and patients

**Figure 1:**
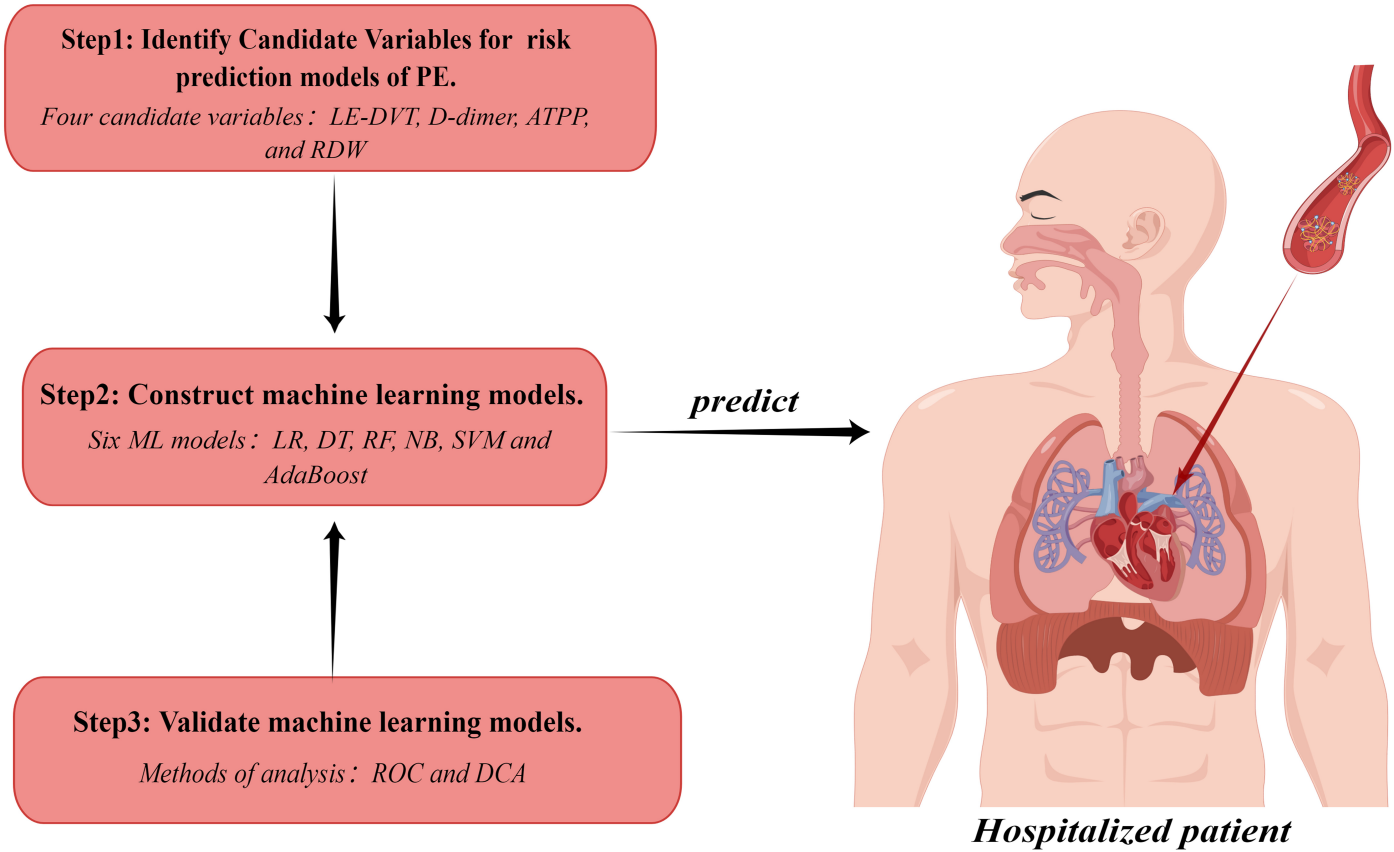
Study workflow chart. PE, pulmonary embolism; LE-DVT, lower extremity deep venous thrombosis; ATTP, activated partial thromboplastin time; RDW, red blood cell distribution width; ML, machine learning; LR, logistic regression; DT, decision tree; RF, random forest; NB, naive bayes; SVM, support vector machine; ROC, receiver operating characteristic curve; DCA, decision curve analysis. The figure was designed by Figdraw (www.figdraw.com).

This study was approved by the Medical Ethics Committee of the Affiliated Hospital of Guangdong Medical University (Ethics Number: YJYS2021172). Figure 1 shows an overview of our study.

In this study, we retrospectively collected the clinical data of all hospitalized patients who underwent CTPA at the Affiliated Hospital of Guangdong Medical University from November 2021 to September 2019. After implementing inclusion and exclusion criteria, a total of 332 patients were screened, resulting in the selection of 172 positive cases and 160 negative cases. The inclusion criteria were as follows: patients aged between 18 and 90 years who had been hospitalized for a minimum period of 3 days; patients with a favorable computed tomographic pulmonary angiography (CTPA) showing a definitive diagnosis or exclusion of PE; and the patients’ medical records (medical history, clinical manifestations, and laboratory results, etc.) are comparatively complete. The exclusion criteria were as follows: patients with a previous history of PE or currently taking medication for its prevention; women during pregnancy.

### Diagnostic criteria of PE

The diagnosis of pulmonary embolism was established through the analysis of the patient’s CTPA, which revealed the presence of filling defects in the pulmonary arteries and their branches. Patients exhibiting filling defects in the pulmonary arteries or any of their branches were categorized as PE-positive. Conversely, patients whose pulmonary arteries appeared suitably opacified without any filling defects were classified as PE-negative (14).

### Data collection and processing

Clinical features and laboratory parameters of all subjects enrolled in the study were retrospectively gathered. Clinical features comprised gender, age, tachypnea, chest pain, cough, hemoptysis, lower extremity pain, hypertension, coronary heart disease, chronic obstructive pulmonary disease (COPD), cancer, recent surgery, atrial fibrillation, cerebrovascular accident, and lower extremity deep vein thrombosis (LE-DVT). The set of laboratory parameters included prothrombin time (PT), activated partial thromboplastin time (APTT), thrombin time (TT), D-dimer, total protein (TP), red blood cell count (RBC), red blood cell distribution width (RDW), platelet count (PLT), platelet distribution width (PDW), albumin, cystatin C, creatinine, and triglycerides.

Data cleaning procedures were executed using R software (version 4.2.1). Initially, features and parameters with more than 5% missing data (cancer, recent surgery, atrial fibrillation, cerebrovascular accident, creatinine, and tri glycerides) were excluded. This resulted in the identification of 11 clinical features (gender, age, tachypnea, chest pain, cough, hemoptysis, lower extremity pain, hypertension, coronary heart disease, COPD, and LE-DVT) and 11 laboratory parameters (PT, APTT, TT, D-dimer, TP, RBC, RDW, PLT, PDW, albumin, and cystatin C). Subsequently, mean values were employed to estimate missing values for continuous variables, while categorical variables, being devoid of deficiencies, were not subject to processing.

### Machine learning models

Using the ML algorithm, six models were developed to predict the risk of PE in hospitalized patients, namely Logistic Regression (LR), Decision Trees (DT), Random Forest (RF), Naive Bayes (NB), Support Vector Machine (SVM), and AdaBoost. The dataset was randomly split into a 70% training group and a 30% independent validation group. During models’ construction, the ten-fold cross-validation method was employed. Specifically, the training group data were randomly partitioned into 10 subsets, with 9 subsets used for models training and 1 for algorithm performance evaluation. This process iterated 10 times to encompass all possible subset combinations, resulting in 10 models. These models were then evaluated on the independent validation group, and mean values of evaluation metrics were computed. The evaluation metrics comprised sensitivity, specificity, area under the curve (AUC), receiver operating characteristic (ROC) curve, and decision curve analysis (DCA). Furthermore, a grid search was conducted to optimize parameters for the ML models.

### Statistical analysis

All statistical tests were implemented based on R 4.2.1. A completely randomized sampling method was employed to allocate 332 patients into a training group and a validation group, with a ratio of 7:3. The Shapiro–Wilk test was used to assess the normality of continuous variables. Normally distributed continuous variables were evaluated using independent samples t-tests and presented as mean ± standard deviation (SD). Non-normally distributed continuous variables were assessed using the Wilcoxon rank sum test and reported as median (Q1, Q3). Categorical variables were analyzed using the chi-square test and presented as numbers (*n*, %). A value of *p* < 0.05 was considered statistically significant. Subsequently, statistically significant variables were subjected to univariate and multivariate logistic regression analyses to determine potential independent risk factors associated with PE. These potential risk factors for PE were employed as predictor variables for the training and evaluating of ML models.

In this study, the “glm()” function was used to perform univariate and multivariate logistic regression analysis, the “caret” package was used to construct ML models, the “ggplot2” package was used to generate ROC curves, and the “dca.r” package was used to plot DCA curves.

## Results

### Baseline characteristics

A total of 332 patients were included in this study. The participants were divided into training and validation groups using complete randomized sampling, with a ratio of 7:3. The training group consisted of 232 patients, including 128 positive cases and 104 negative cases. The validation group consisted of 100 patients, with 44 positive cases and 56 negative cases. In the training group dataset, statistically significant differences were observed between PE-positive and PE-negative patients (*P* < 0.05) in terms of lower extremity pain, LE-DVT, D-dimer, ATPP, RDW, tachypnea, and cough. However, no statistically significant differences were found between PE-positive patients and PE-negative patients in terms of gender, age, chest pain, hemoptysis, hypertension, coronary heart disease, COPD, PT, TT, RBC, PLT, PDW, TP, albumin, and cystatin C (*P* > 0.05). The baseline characteristics of the patients are presented in Table 1.

**Table 1:** Baseline characteristics of the patients.

Variables	Training group (*n* = 232)			Validation group (*n* = 100)		*P*
	PE positive (*n* = 128)	PE negative (*n* = 104)	*P*	PE positive (*n* = 44)	PE negative (*n* = 56)	
Gender, *n* (%)			0.974			0.482
Male	70 (54.7)	58 (55.8)		26 (59.1)	28 (50.0)	
Female	58 (45.3)	46 (44.2)		18 (40.9)	28 (50.0)	
Tachypnea, *n* (%)			0.009			0.546
Yes	58 (45.3)	66 (63.5)		20 (45.5)	30 (53.6)	
No	70 (54.7)	38 (36.5)		24 (54.5)	26 (46.4)	
Chest pain, *n* (%)			0.117			0.034
Yes	61 (47.7)	38 (36.5)		19 (43.2)	12 (21.4)	
No	67 (52.3)	66 (63.5)		25 (56.8)	44 (78.6)	
Cough, *n* (%)			0.022			0.070
Yes	51 (39.8)	58 (55.8)		17 (38.6)	33 (58.9)	
No	77 (60.2)	46 (44.2)		27 (61.4)	23 (41.1)	
Hemoptysis, *n* (%)			0.951			0.692
Yes	10 (7.8)	7 (6.7)		2 (4.5)	4 (7.1)	
No	118 (92.2)	97 (93.3)		42 (95.5)	52 (92.9)	
Lower extremity pain, *n* (%)			0.037			0.235
Yes	14 (10.9)	3 (2.9)		5 (11.4)	2 (3.6)	
No	114 (89.1)	101 (97.1)		39 (88.6)	54 (96.4)	
LE-DVT, *n* (%)			0.003			0.006
Yes	15 (11.7)	1 (1.0)		6 (13.6)	0 (0.0)	
No	113 (88.3)	103 (99.0)		38 (86.4)	56 (100.0)	
Hypertension, *n* (%)			0.359			0.908
Yes	44 (34.4)	29 (27.9)		12 (27.3)	17 (30.4)	
No	84 (65.6)	75 (72.1)		32 (72.7)	39 (69.6)	
Coronary heart disease, *n* (%)			0.709			0.052
Yes	18 (14.1)	12 (11.5)		10 (22.7)	4 (7.1)	
No	110 (85.9)	92 (88.5)		34 (77.3)	52 (92.9)	
COPD, *n* (%)			0.599			0.206
Yes	11 (8.6)	12 (11.5)		7 (15.9)	4 (7.1)	
No	117 (91.4)	92 (88.5)		37 (84.1)	52 (92.9)	
Age (years), median (Q1, Q3)	70.00 (55.00, 80.00)	66.00 (57.00, 73.25)	0.159	65.50 (53.75, 76.25)	63.50 (50.75, 78.00)	0.581
D-dimer (mg/L), median (Q1, Q3)	7.62 (3.65, 14.84)	2.71 (1.12, 7.41)	<0.001	7.78 (4.08, 13.60)	2.34 (0.94, 9.18)	0.001
PT (s), median (Q1, Q3)	13.00 (12.30, 13.85)	13.40 (12.40, 14.33)	0.250	13.50 (12.40, 15.03)	13.50 (12.70, 15.33)	0.434
APTT (s), median (Q1, Q3)	32.65 (27.73, 38.43)	35.70 (31.50, 39.60)	0.005	33.85 (29.80, 37.43)	35.35 (32.63, 40.90)	0.025
TT (s), median (Q1, Q3)	17.00 (15.20, 18.20)	17.15 (16.08, 18.70)	0.148	16.90 (15.68, 18.48)	17.10 (16.08, 18.60)	0.569
RBC (*10^9 ^/L), Mean ± SD	4.13 ± 0.70	3.99 ± 0.78	0.141	3.96 ± 0.87	3.82 ± 0.77	0.418
RDW (%), median (Q1, Q3)	13.90 (12.98, 15.10)	13.20 (12.60, 14.00)	<0.001	15.15 (13.48, 16.93)	13.65 (13.10, 15.23)	0.038
PLT (*10^9 ^/L), median (Q1, Q3)	226.50 (174.00, 292.50)	228.00 (176.75, 317.00)	0.704	208.00 (155.25, 272.25)	241.50 (152.50, 278.00)	0.652
PDW (%), median (Q1, Q3)	12.20 (10.40, 16.30)	12.00 (9.80, 15.63)	0.115	15.30 (11.68, 16.38)	11.15 (9.88, 15.73)	0.001
TP (g/L), mean ± SD	61.99 ± 7.88	62.73 ± 7.82	0.480	62.97 ± 7.87	62.11 ± 9.56	0.622
Albumin (g/L), median (Q1, Q3)	35.85 (30.48, 39.32)	36.15 (30.18, 39.08)	0.944	35.65 (31.70, 38.05)	36.30 (31.20, 39.65)	0.857
Cystatin C(mg/L), median (Q1, Q3)	0.88 (0.72, 1.03)	0.86 (0.75, 1.08)	0.277	0.98 (0.80, 1.10)	0.94 (0.78, 1.08)	0.378

PE, pulmonary embolism; COPD, chronic obstructive pulmonary disease; LE-DVT, lower extremity deep venous thrombosis; PT, prothrombin time; APTT, activated partial thromboplastin time; TT, thrombin time; RBC, blood cell count; RDW, red blood cell distribution width; PLT, platelet count; PDW, platelet distribution width; TP, total protein.

### Logistic regression analysis

Based on the baseline characteristics of the patients, we conducted screening of seven variables (tachypnea, cough, lower extremity pain, LE-DVT, D-dimer, ATPP, and RDW) that exhibited statistical significance (*P* < 0.05) in the training group. Subsequently, these variables were included in both univariate and multivariate logistic regression analyses. The univariate logistic regression analysis identified tachypnea, cough, lower extremity pain, LE-DVT, D-dimer, ATPP, and RDW as potential influencing factors for PE (*p* < 0.05, Table 2), which aligns with the findings presented in Table 1. To eliminate the effect of confounding factors, we incorporated the influencing factors of PE into the multivariate logistic regression analysis. The results of this analysis indicated that LE-DVT, elevated D-dimer levels, shortened ATPP, and increased RDW may be potential independent risk factors for PE patients (*p* < 0.05, Table 2).

**Table 2:** Univariate and multivariate logistic regression analysis.

Variables	Univariate analysis			Multivariate analysis		
	OR	95% CI	*p*-value	OR	95% CI	*p*-value
Tachypnea	0.477	0.279–0.807	0.006	0.761	0.416–1.395	0.376
Cough	0.525	0.310–0.885	0.016	0.68	0.378–1.221	0.196
Lower extremity pain	4.135	1.304–18.306	0.029	1.251	0.312–6.231	0.762
LE-DVT	13.673	2.699–249.410	0.012	12.001	2.160–225.754	0.021
D-dimer	1.036	1.012–1.067	0.01	1.029	1.004–1.061	0.042
ATPP	0.952	0.921–0.982	0.003	0.951	0.918–0.983	0.004
RDW	1.196	1.053–1.382	0.01	1.206	1.043–1.417	0.016

PE, pulmonary embolism; OR, odds ratio; CI, confidence interval; LE-DVT, lower extremity deep venous thrombosis; ATPP, activated partial thromboplastin time; RWD, red blood cell distribution width.

### Construction and validation of machine learning models

**Figure 2:**
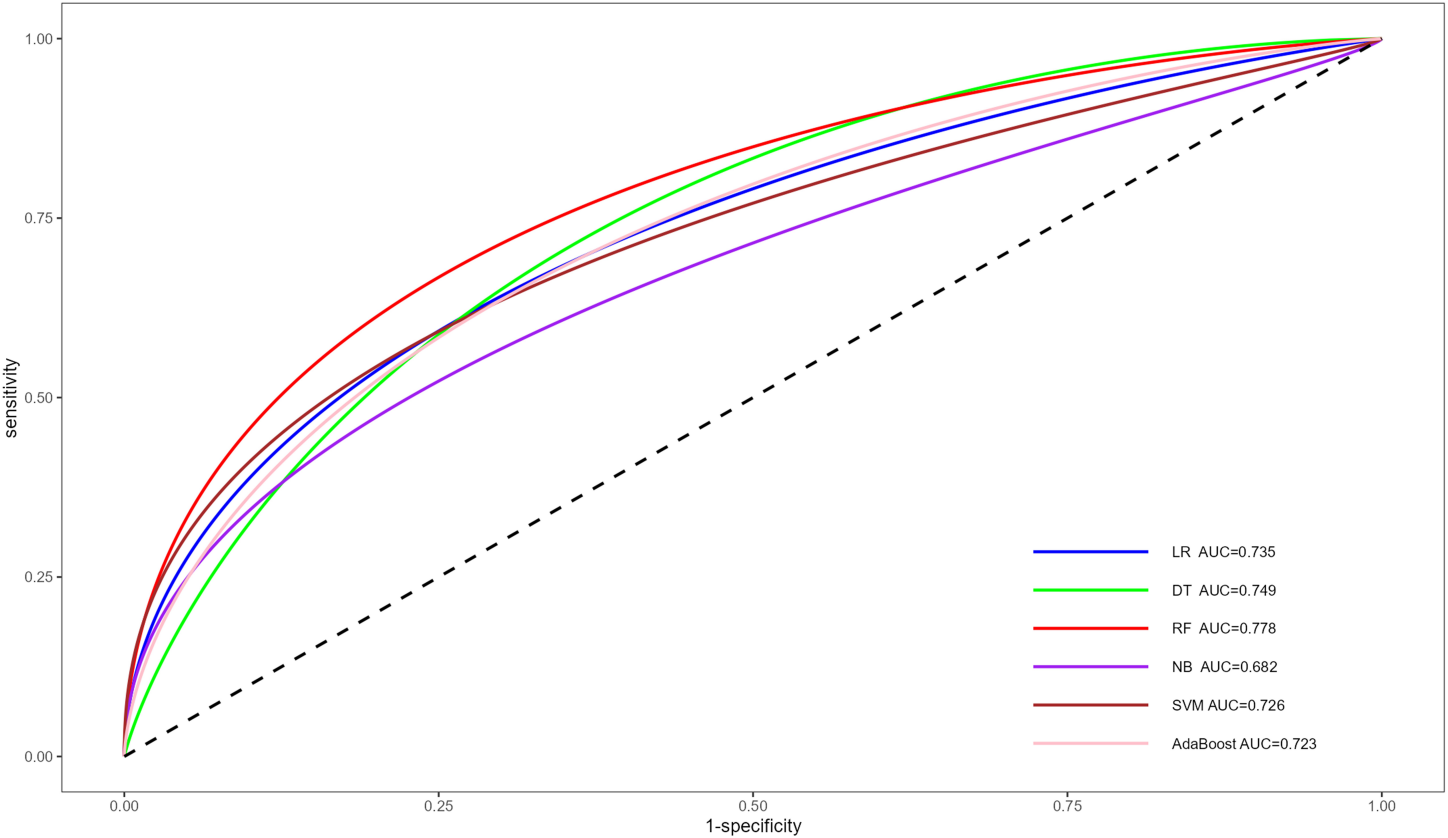
ROC for the prediction of PE risk of different ML models. ROC, receiver operating characteristic curve; PE, pulmonary embolism; ML, machine learning; AUC, area under the curve; LR, logistic regression; DT, decision tree; RF, random forest; NB, naive bayes; SVM, support vector machine.

**Figure 3:**
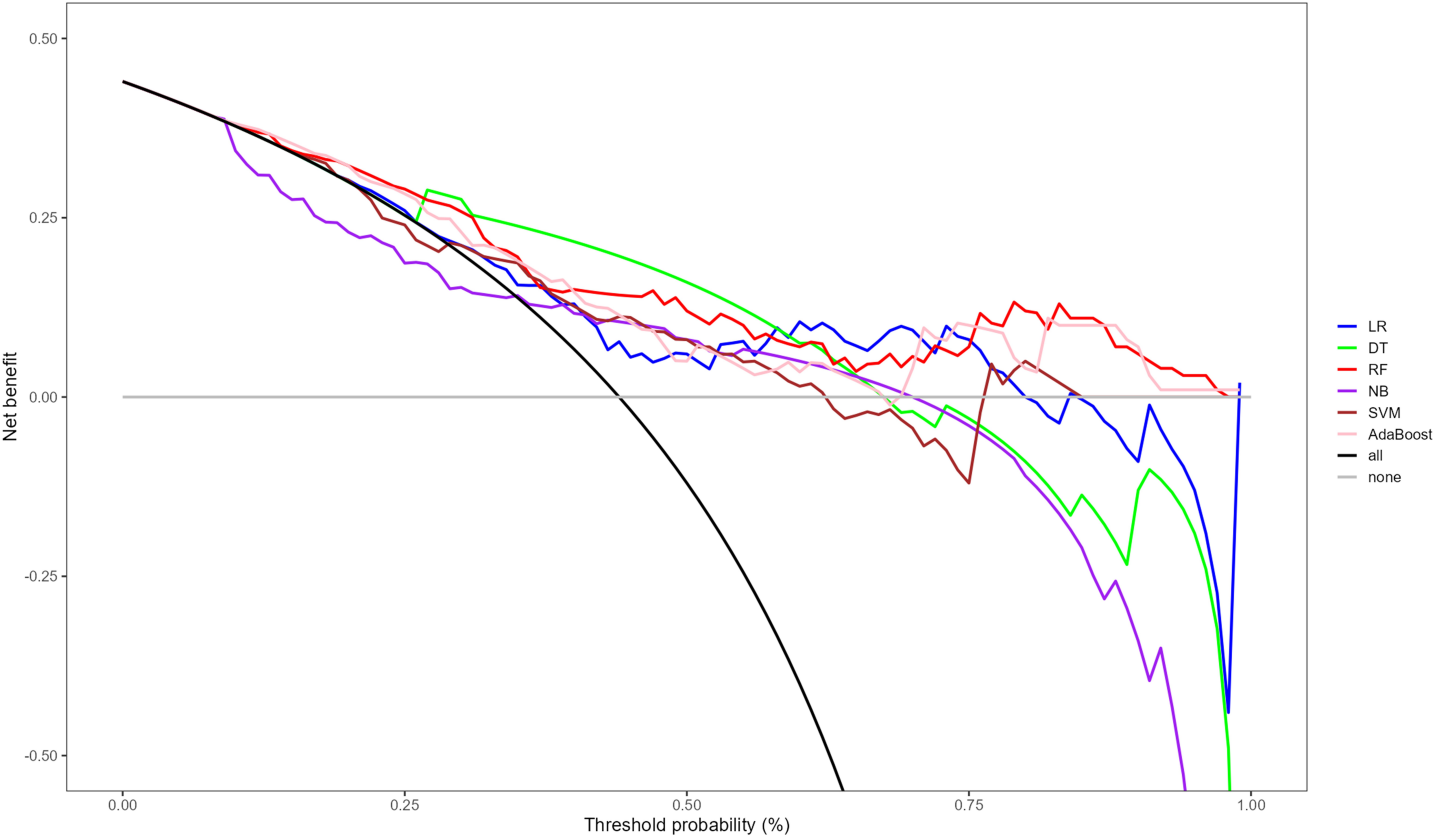
DCA for the prediction of PE risk of different ML models. DCA, decision curve analysis; PE, pulmonary embolism; ML, machine learning; LR, logistic regression; DT, decision tree; RF, random forest; NB, naive bayes; SVM, support vector machine.

**Figure 4:**
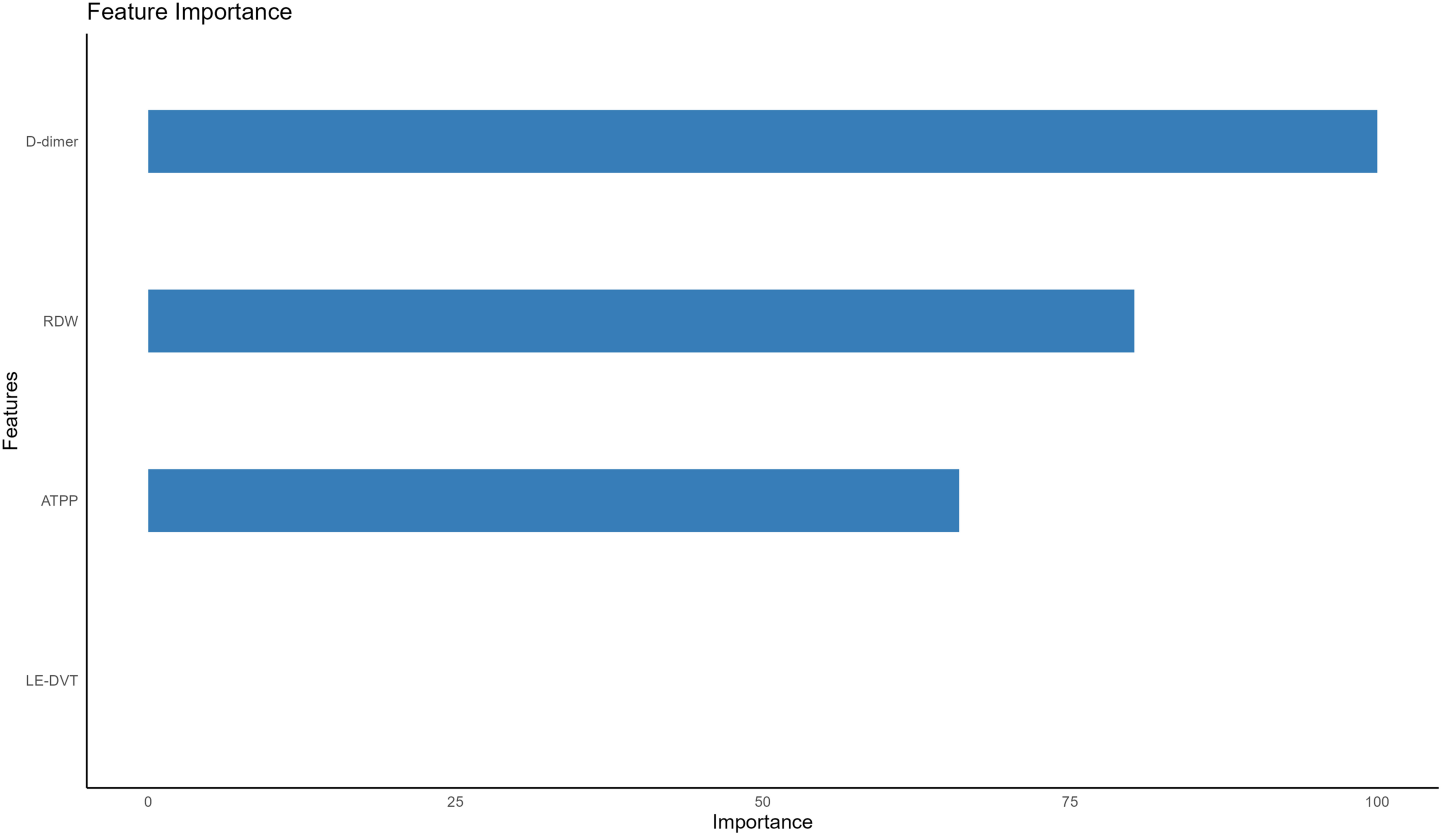
Importance of the four predictors. LE-DVT, lower extremity deep venous thrombosis; RDW, red blood cell distribution width; ATPP, activated partial thromboplastin time.

Following multivariate logistic regression screening, four potential independent risk factors (LE-DVT, D-dimer, ATPP, and RDW) among PE patients were identified as predictors for constructing six ML models, whose performance was evaluated on the validation dataset. Table 3 presents four metrics for assessing model performance, including AUC with 95% confidence intervals, sensitivity, specificity, and accuracy. The AUC, sensitivity, specificity, and accuracy of these ML models were LR (0.735, 75.0%, 51.8%, 62.0%), DT (0.749, 75.0%, 69.6%, 72.0%), RF (0.778, 77.3%, 60.7%, 68.0%), NBC (0.682, 34.1%, 87.5%, 64%), SVM (0.726, 75%, 55.4%, 64%), and AdaBoost (0.723, 65.9%, 57.1%, 61%) (Table 3). Notably, RF demonstrated the most robust predictive performance among these ML models, exhibiting the highest AUC of 0.778. This finding was further corroborated by ROC analysis, as depicted in Figure 2. Furthermore, DCA analysis illustrated that RF yielded the highest net benefit across most risk thresholds, thereby affirming its superiority (Figure 3). Figure 4 depicts the importance of predictor variables in the RF model, with D-dimer exerting the greatest impact, followed by RDW, ATPP, and LE-DVT.

**Table 3:** Predictive efficacy analysis of different ML models for PE.

	AUC (95% CI)	Sensibility	Specificity	Accuracy
LR	0.735 (0.633–0.837)	75.0%	51.8%	62.0%
DT	0.749 (0.651–0.846)	75.0%	69.6%	72.0%
RF	0.778 (0.686–0.870)	77.3%	60.7%	68.0%
NB	0.682 (0.572–0.791)	34.1%	87.5%	64.0%
SVM	0.726 (0.622–0.830)	75.0%	55.4%	64.0%
AdaBoost	0.723 (0.623–0.823)	65.9%	57.1%	61.0%

ML, machine learning; PE, pulmonary embolism; LR, logistic regression; DT, decision tree; RF, random forest; NB, naive bayes; SVM, support vector machine; AUC, area under the curve.

## Discussion

In this study, we developed six ML models for predicting the occurrence of PE in hospitalized patients. The training group dataset was utilized to construct these models, and their efficacy was assessed using the independent validation group dataset. The careful selection of candidate variables plays a crucial role in constructing accurate prediction models for PE occurrence in hospitalized patients. Initially, we screened seven statistically significant candidate variables by comparing baseline characteristics of PE-positive and PE-negative patients. To assess the significance of these variables, univariate logistic regression analysis was employed. Furthermore, multivariate logistic regression was utilized to address potential confounding factors and refine the selection of candidate variables. Finally, integrating findings from existing studies and clinical practices, the pool of candidate variables was narrowed down to four: LE-DVT, D-dimer, ATPP, and RDW. These four variables were then incorporated into the construction of our PE risk prediction models.

Our study demonstrated that patients with combined LE-DVT have a 12.001-fold higher risk of developing PE compared to patients without combined LE-DVT. This finding emphasizes LE-DVT as an risk factor for the development of PE, supporting previous reports (15).

Plasma D-dimer, a specific fibrin degradation product, serves as a reliable marker for thrombus formation (16). According to Schutte et al., elevated D-dimer levels are considered a risk factor for PE (17). Alexander's study indicated that D-dimer levels exceeding 2,000 μg/L are associated with a 33.9% risk of PE, further confirming high D-dimer levels as an independent risk factor for PE (18). Consistent with the aforementioned study, our research revealed that elevated levels of D-dimer may increase the risk of PE.

APTT serves as a comprehensive coagulation screening test, sensitive to deficiencies in factors II, V, VIII, IX, X, XI, XII, and fibrinogen (19, 20). Studies have demonstrated that decreased APTT levels are associated with an elevated risk of thrombosis, potentially attributed to an altered coagulation mechanism resulting from increased coagulation factor activity (21). Armando's study independently correlated hypercoagulability, detected by shortened APTT, with the risk of VTE (22). According to Zakai et al., the cumulative incidence of VTE over a 13-year follow-up period is correlated with APTT levels, with shortened APTT increasing the risk of future VTE (23). Corresponding to the aforementioned studies, our study revealed that APTT shortening may increase the risk of PE.

Furthermore, our study demonstrated that increased RDW may be a risk factor for PE, consistent with related studies (24). RDW, a simple parameter derived from blood routine, reflects the heterogeneity of circulating red blood cell size (25). Multiple studies have confirmed the association between RDW and the severity, prognosis, and predictive value of PE (26–28). Additionally, elevated RDW levels serve as an independent predictor of early PE-related mortality (29). The potential mechanism for the association between RDW and PE could involve the relationship between RDW, acute inflammatory markers, and alterations in blood viscosity (26). However, this mechanism has not been extensively investigated and requires further detailed research.

In conclusion, there exists a close association between LE-DVT, D-dimer, ATPP, and RDW with PE. Therefore, it is justifiable to incorporate these variables in the development of a predictive model for assessing the risk of PE.

In this study, we employed six common supervised machine learning methods, namely LR, DT, RF, NB, SVM, and AdaBoost, to construct risk prediction models. These methods are widely used in data mining and analysis, as well as for the prediction of various diseases. For instance, LR has been employed for the prediction of liver disease (30), DT for heart disease (31), RF for diabetes (32), NB for cardiovascular disease (33), SVM for hypertension (34), and AdaBoost for Parkinson's disease (35). LR is a classification model that calculates the probability of PE occurrence in patients by fitting a Sigmoid Function to the input sample data. SVM projects data from all patients onto a higher-dimensional feature space and creates a decision boundary to differentiate between PE-positive and PE-negative patients. NB independently learns the distribution of each variable in the PE-positive and PE-negative cohorts, allowing it to calculate the probability of PE in each patient. DT is a tree-structured classifier that makes decisions by asking binary questions. The construction process of a DT begins at the root node and continues through the internal nodes, where binary questions are posed. The algorithm selects the next branch of the tree based on the answer to the question (either yes or no), progressing closer to a leaf node. The root node represents the entire sample, while the branches depict decision rules, and the leaf nodes indicate the decision outcomes (36, 37). Both RF and AdaBoost are ensemble learning methods that aim to create robust classifiers by combining multiple weak classifiers, thereby enhancing the accuracy of predictions. However, they employ different training mechanisms. RF employs the bagging algorithm to independently construct weak classifiers, whereas AdaBoost, based on the boosting algorithm, involves an iterative training process for weak classifiers. In AdaBoost, the results of previous weak classifiers influence the construction of subsequent weak classifiers (38, 39).

The impact of parameters on the performance of ML models is widely acknowledged. To mitigate this influence and optimize the models to the fullest extent possible, a grid search approach was employed during their construction. Through this method, we systematically explored the parameter space to identify the optimal parameters or most effective parameter combinations for each ML model (40). For instance, DT parameter was set to cp = 0.01, RF parameter to mtry = 1, the parameter combinations for NB included fL = 0.1, usekernel = False, and adjust = 0.1, while for SVM, parameters were set to sigma = 0.5 and C = 0.1. Similarly, AdaBoost parameter combinations were defined as mfinal = 10, maxdepth = 30, and coeflearn = Freund. However, for LR, grid search was not conducted, as the “caret” package lacks parameters for LR. Notably, the parameters or parameter combinations offered by the “caret” package for the ML models are not adequately comprehensive, potentially limiting the scope for achieving greater optimization or tuning of the models.

Through the analysis of ROC and AUC, we observed that the RF model outperformed other models by achieving the highest AUC of 0.778, indicating superior predictive efficacy. The superior performance of the RF model in this context may be attributed to its compatibility with relatively small sample sizes, as well as its inherent advantages such as stability, ability to handle unbalanced datasets, and capability to reduce overfitting (41, 42). Furthermore, the results of the DCA revealed that the RF model consistently achieved the highest net benefit across the majority of threshold intervals, providing further confirmation of its superior performance over other models.

This study presents several advantages. Firstly, the ML models developed in this study have been specifically designed for inpatients and exhibit a high level of accuracy in predicting the risk of PE. While certain scoring rules, like the commonly used Well score and revised Geneva score, have been developed to assess the risk of PE, their validation has predominantly been limited to outpatients suspected of having PE, and they have not been adequately validated in the inpatient population (43). In fact, another study has emphasized that the application of the Well score in inpatients is inappropriate (6). Although clinical predictive scores such as the Caprine score (44), Padua score (45), and IMPROVE score (46) can be utilized to determine the risk of VTE in hospitalized patients, they are not comprehensive scoring tools specific to PE risk assessment. Therefore, relying on these scoring rules to predict the risk of PE in hospitalized patients is considered unreliable. On the contrary, our ML models have been developed and evaluated using a broad spectrum of hospitalized patients, confirming their efficacy, and demonstrating a high level of accuracy in predicting the risk of PE in hospitalized patients.

Secondly, given the high accuracy, user-friendly nature, and cost-effectiveness of the ML models we have developed, they are expected to play a promising auxiliary role in the detection of PE.

Lastly, this study stands out by developing multiple ML models. Previous studies have often employed a single ML method for modeling without providing a clear comparison of the advantages of different modeling methods. In contrast, we have trained multiple machine learning models and compared their efficacy and clinical benefits. This approach informs subsequent studies on the development of risk prediction models for PE in hospitalized patients, enabling the selection of the most suitable model.

Nonetheless, this study possesses several limitations. Firstly, all cases included in this study originated solely from a single hospital, thereby lacking data from diverse populations or other healthcare organizations for external validation. Secondly, the sample size of the included study is limited, and the clinical data of hospitalized patients is insufficient. Thirdly, it is a retrospective study and requires extensive prospective validation.

## Conclusion

In this study, we identified several potential independent risk factors for PE, including EL-DVT, elevated D-dimer levels, shortened ATPP, and increased RDW. Subsequently, we developed six ML models to predict the occurrence of PE in hospitalized patients. Among these models, the RF model demonstrated the highest predictive efficacy and clinical benefit in accurately identifying and forecasting the risk of PE in hospitalized patients.

## Data Availability

The raw data supporting the conclusions of this article will be made available by the authors, without undue reservation.
